# Deep-learning enabled combined measurement of tumour cell density and tumour infiltrating lymphocyte density as a prognostic biomarker in colorectal cancer

**DOI:** 10.1038/s44276-025-00123-8

**Published:** 2025-03-03

**Authors:** Alice C. Westwood, Benjamin I. Wilson, Jon Laye, Heike I. Grabsch, Wolfram Mueller, Derek R. Magee, Phillip Quirke, Nicholas P. West

**Affiliations:** 1 Division of Pathology and Data Analytics, Leeds Institute of Medical Research at St James’s, University of Leeds, Leeds, UK; 2 School of Computing, University of Leeds, Leeds, UK; 3 Department of Pathology, GROW – Research Institute for Oncology and Reproduction, Maastricht University Medical Center+, Maastricht, Netherlands; 4 Gemeinschaftspraxis Pathologie, Starnberg, Germany

## Abstract

**Background:**

Within the colorectal cancer (CRC) tumour microenvironment, tumour infiltrating lymphocytes (TILs) and tumour cell density (TCD) are recognised prognostic markers. Measurement of TILs and TCD using deep-learning (DL) on haematoxylin and eosin (HE) whole slide images (WSIs) could aid management.

**Methods:**

HE WSIs from the primary tumours of 127 CRC patients were included. DL was used to quantify TILs across different regions of the tumour and TCD at the luminal surface. The relationship between TILs, TCD, and cancer-specific survival was analysed.

**Results:**

Median TIL density was higher at the invasive margin than the luminal surface (963 vs 795 TILs/mm^2^, *P* = 0.010). TILs and TCD were independently prognostic in multivariate analyses (HR 4.28, 95% CI 1.87–11.71, *P* = 0.004; HR 2.72, 95% CI 1.19–6.17, *P* = 0.017, respectively). Patients with both low TCD and low TILs had the poorest survival (HR 10.0, 95% CI 2.51–39.78, *P* = 0.001), when compared to those with a high TCD and TILs score.

**Conclusions:**

DL derived TIL and TCD score were independently prognostic in CRC. Patients with low TILs and TCD are at the highest risk of cancer-specific death. DL quantification of TILs and TCD could be used in combination alongside other validated prognostic biomarkers in routine clinical practice.

## Background

Colorectal cancer (CRC) is the third most commonly diagnosed malignancy globally and is the second most common cause of cancer death with over 44,000 new cases and over 16,800 deaths every year in the United Kingdom [1, 2]. In routine clinical practice, disease stage, as determined by the Union for International Cancer Control tumour node metastasis (TNM) system, is primarily used to predict CRC patient prognosis and guide treatment decisions along with other validated adverse pathological features [3–5]. However, patients diagnosed at the same disease stage may have differing outcomes [6, 7] and 20–50% of patients initially diagnosed with localised disease, will ultimately develop distant metastases [8]. There is therefore an urgent need for novel validated prognostic biomarkers that are reproducible, have a short turnaround time, and are easy to implement into routine pathology workflow, to further stratify patients who may benefit from adjuvant therapy or who may be cured by surgery alone.

Tumour infiltrating lymphocytes (TILs) are a major component of the tumour microenvironment and high numbers of TILs have been associated with improved survival in CRC and other tumour types [9–11]. There are several existing TIL classification systems that rely on the subjective quantification of the lymphocytic infiltration or the scoring of lymphoid aggregates or “Crohn’s-like lymphoid reaction” [10–12]. However, manually scoring TILs using the aforementioned systems is limited by high inter-observer variability, is time-consuming and therefore hasn’t been implemented into routine clinical practice [13, 14]. Ourselves and others, including Galon et al., have recognised that using immunohistochemistry (IHC) to quantitate the density of T-cell subsets is important, however, this remains expensive and time consuming to perform, hindering its use in routine clinical practice [15–18]. It is also important to note that quantification of TILs on haematoxylin and eosin (HE) slide includes the assessment of all lymphocytes (T and B cells), whereas IHC for T-cell subsets only assesses T cells, so values may differ between techniques. To date, quantification of TILs has focused on assessing the deep invasive margin (e.g. Jass score) and/or the central tumour region in resection specimens [9]. Little attention has been paid to the luminal surface of the tumour, despite this being the only area sampled in endoscopic biopsies. Although using IHC to explore T cell subsets has been performed in rectal biopsies, there has been minimal focus on quantification of TILs on HE in biopsies [19–21]. With increasing use of neoadjuvant treatment in CRC patients, biomarkers applied to routine endoscopic biopsy samples will play a key role in prognostication and prediction of response to treatment in the future.

An alternative method of quantifying TILs, without the need for IHC, is the use of deep-learning (DL) on a high resolution digitally scanned HE stained slide. The benefits of using DL in this capacity would be two-fold, in the clinical setting it would be cheap, rapid and eliminate pathologist inter-observer variability and in the research setting it would allow the analysis of large datasets quickly to generate robust data. DL detection of TILs on HE has been shown to be prognostic, with a high number of TILs associated with a favourable prognosis in a range of cancer types including breast cancer [22, 23], gastric cancer [24], and melanoma [25].

The number of lymphocytes also differs depending on the specific region of the tumour that has been assessed; several studies have found that the number of lymphocytes was greater at the invasive margin compared to the central tumour [11, 26, 27]. In these assessments, the luminal surface has always been incorporated into the “central tumour” region, and hasn’t been separately investigated.

In addition to TILs, the relative proportion of malignant epithelial cells and stroma, known as tumour cell density (TCD) or tumour-stroma ratio, has been identified as a prognostic factor in patients with potentially curative CRC with a high proportion of stroma associated with poorer survival [28–33]. As with TILs, TCD has been calculated in different ways between studies, with the majority being conducted manually either using a subjective estimation or an objective point counting technique at the luminal surface or invasive margin [28–31, 34]. More recently, TCD estimation has been carried out using DL [35, 36]. The benefit of combining TCD and TIL scores has recently been demonstrated through the Glasgow Microenvironment Score which is a prognostic marker in stage I-III CRC, however this system requires manual assessment of both TCD and TILs and neither marker was measured at the luminal surface [37]. DL measurement of TILs and TCD would have the benefit of being quicker, cheaper, allow analysis of large datasets, and potentially more objective and convenient compared to manual measurement.

We hypothesised that: (a) high DL-derived TIL density is associated with improved cancer-specific survival on both univariate and multivariate analysis, (b) combining DL-derived TIL and TCD values improves prognostication in CRC, (c) DL-derived TILs and TCD values assessed at the luminal surface could be used to inform neoadjuvant treatment decisions.

## Methods

### Patients and clinicopathological data

Patients who underwent potentially curative surgery for CRC at the Marienhospital, Düsseldorf, Germany between 1^st^ January 1990 and 31^st^ December 1995, were included in this retrospective study. None of the patients received pre-operative chemotherapy or radiotherapy. Patients undergoing palliative surgery were not included in this study. One representative HE stained tumour tissue section from the surgical resection specimen was scanned at x40 magnification (Aperio XT, Aperio Technologies, Vista, CA, USA). Only those with manually derived TCD values from a previous study were included (*n* = 144) [28]. Cases were excluded if the slides did not contain definite invasive adenocarcinoma or the full thickness of the primary tumour; slides with significant artefact were also excluded. Histopathological data were available for all patients including site of tumour, lymphovascular invasion status, maximum depth of invasion (pT category), lymph node involvement (pN category) and distant metastasis (pM category) according to TNM 5^th^ edition [38]. In addition, mismatch repair status was available for all patients from a previous study [39].

This study conforms to the REMARK guidelines (refer to Supplementary Table S1) [40].

### Annotation of tumour regions

Digital slides were annotated using HeteroGenius-MIM image analysis software (HeteroGenius Ltd., Leeds, UK). In each case, three tumour regions were annotated manually by a pathologist (AW): (1) the whole invasive tumour area, including tumour and associated stroma; (2) a 2 mm deep strip at the luminal surface of the tumour, and (3) a 1 mm strip at the deep invasive margin of the tumour (Supplementary Fig. S1). Regions of ulcer slough or necrosis visible at low magnification were excluded from the annotation areas. Areas of dysplasia at the edges of the cancer were excluded from the annotation.

### Image analysis pipeline to establish TIL density

For the detection of TILs, we used our published method [24] which classified cells into 9 different types: lymphocyte, plasma cell, granulocyte, fibroblast, endothelial cell, muscle, tumour, and normal epithelium [24]. This published model had a further ~55,000 annotated cells from a mixture of oesophagogastric cancer cases and colorectal cancer cases added and was trained for a further 9000 epochs. We developed a convolutional neural network model to differentiate between genuine tissue and background space. This model was trained using 147 tissue annotations and 159 background annotations for 2049 epochs. Manual quality control was performed as previously described in three independent regions [24]. TIL density was calculated by dividing the total number of lymphocytes detected within the region of interest by the genuine tissue area of the region of interest. No attempt was made to distinguish between TILs located in the stroma and TILs overlying tumour epithelium [24]. For an example of TILs segmentation in HE stained colorectal cancer, see Supplementary Fig. S2A, B.

### Image analysis pipeline to establish TCD

The annotation previously used to determine TCD by manual point counting (9 mm^2^ region of interest placed at the luminal surface in the region of apparent highest TCD) [28] was uploaded to HeteroGenius-MIM image analysis software. None of the manual spot counting data was used to train the TCD model.

A convolutional neural network derived from a UNET framework [41] was trained using manual point-counting data from 300 +/− 15 points spread equidistant across the whole invasive tumour area from 101 chemo(radio)therapy naive rectal cancers from an independent series; this series was split 60% and 40% for training and testing.

The trained model was then applied to the 9 mm^2^ region of interest at the luminal surface in the current dataset. TCD was calculated by dividing tumour points by all informative points to give a percentage TCD. For an example of TCD segmentation in HE stained colorectal cancer, see Supplementary Fig. S2C, D.

### Statistical analyses

The relationship between TIL density in different regions of the tumour and clinicopathological variables was analysed using the Kruskal–Wallis test. Differences in TIL density between tumour regions were firstly analysed using the Kruskal–Wallis test; if significant, Dunn’s Test with Bonferroni correction was performed to determine the statistical differences between each region. The Pearson correlation coefficient was calculated to analyse correlation between TIL density and TCD.

Cut-off values to dichotomise TCD and TIL density in each region into high or low were based on the best hazard ratio for survival. Dichotomised TCD at the luminal surface and TIL density at the invasive margin were combined to create four groups. The same categorisation was also performed when TIL density was measured at the luminal surface, rather than the invasive margin.

The primary study endpoint was cancer-specific survival, which was available for all patients. Patients who died within 30 days of surgery were excluded from the study. The median follow-up time was 5.3 years and 98 patients (77.2%) were alive at the end of the study period. The relationship of TIL density and TCD with cancer-specific survival was analysed using the Kaplan–Meier method, log-rank test and Cox proportional hazards models [42]. In multivariate analyses, factors found to be significant in univariate survival analysis were included. *P*-values < 0.05 were considered statistically significant.

Statistical analyses were performed using IBM SPSS Statistics (Version 29.0.0.0, IBM, Armonk, NY, USA), R, version 4.2.2 (R Foundation for Statistical Computing, Vienne, Austria), and python version 3.8. R packages used were: *ggplot2* (version 3.4.4)*, ggpubr* (version 0.6.0), *FSA* (version 0.9.5), *survival* (version 3.5-3), *survminer* (version 0.4.9), and *dplyr* (version 1.1.0); python packages used were *lifelines* (version 0.28.0).

## Results

### TIL density in different tumour regions

Of the 144 cases initially identified, 17 were excluded (Supplementary Fig. S3). 127 CRC cases were therefore included in the final analysis. The median (range) area of each annotation was 94.0 mm^2^ (22.1–439.3 mm^2^) in the whole invasive tumour, 18.5 mm^2^ (3.0–52.2 mm^2^) at the invasive margin, and 32.6 mm^2^ (7.2–109.8 mm^2^) at the luminal surface.

The median (range) TIL density varied between the different tumour regions; whole tumour 810 TILs/mm^2^ (221–3404 TILs/mm^2^), invasive margin 963 TILs/mm^2^ (296–3432 TILs/mm^2^), and luminal surface 795 TILs/mm^2^ (range: 259–3471 TILs/mm^2^). TIL density at the invasive margin was significantly greater than that at the luminal surface (*P* = 0.010) and across the whole tumour area (*P* = 0.015). TIL density in any of the regions measured was not significantly related to any clinicopathological variables (Table 1), including MMR status.

**Table 1:** Relationship between TIL density in different regions of the tumour and clinicopathological variables.

	All cases		Invasive margin TIL density (mm^2^)	Whole tumour area TIL density (mm^2^)	Luminal surface TIL density (mm^2^)
	*n*	%	*p*-value	*p*-value	*p*-value
**Sex**					
Male	50	39.4	0.953	0.730	0.661
Female	77	60.6			
**Age (years)**					
<65	42	33.1	0.697	0.975	0.716
≥65	85	66.9			
**Tumour location**					
Colon	89	70.1	0.111	0.052	0.070
Rectum	38	29.9			
**Adjuvant chemotherapy**					
No	107	84.3	0.848	0.791	0.968
Yes	20	15.7			
**pT category**^**a**^					
pT1/pT2	31	24.4	0.632	0.258	0.208
pT3	87	68.5			
pT4	9	7.1			
**pN category**^**a**^					
pN0	83	65.4	0.158	0.266	0.596
pN1	28	22.0			
pN2	16	12.6			
**TNM stage**^**a**^					
I	26	20.5	0.435	0.218	0.213
II	56	44.1			
III	44	34.6			
IV	1	0.8			
**Distant metastasis**					
No	126	99.2	0.626	0.623	0.978
Yes	1	0.8			
**Lymphovascular invasion**					
No	122	96.1	0.465	0.488	0.413
Yes	5	3.9			
**Mismatch repair status**					
pMMR	106	83.5	0.186	0.426	0.468
dMMR	17	13.4			
NK	4	3.1			

*P*-values that are statistically significant are shown in bold.

*pT* depth of invasion, *pN* lymph node status, *pMMR* proficient mismatch repair, *dMMR* deficient mismatch repair, *NK* not known, *TIL* tumour infiltrating lymphocytes.

^a^Tumour-Node-Metastasis stage grouping, pT category and pN category were obtained using TNM, 5^th^ edition [38].

### TIL density and TCD and survival

**Fig. 1:**
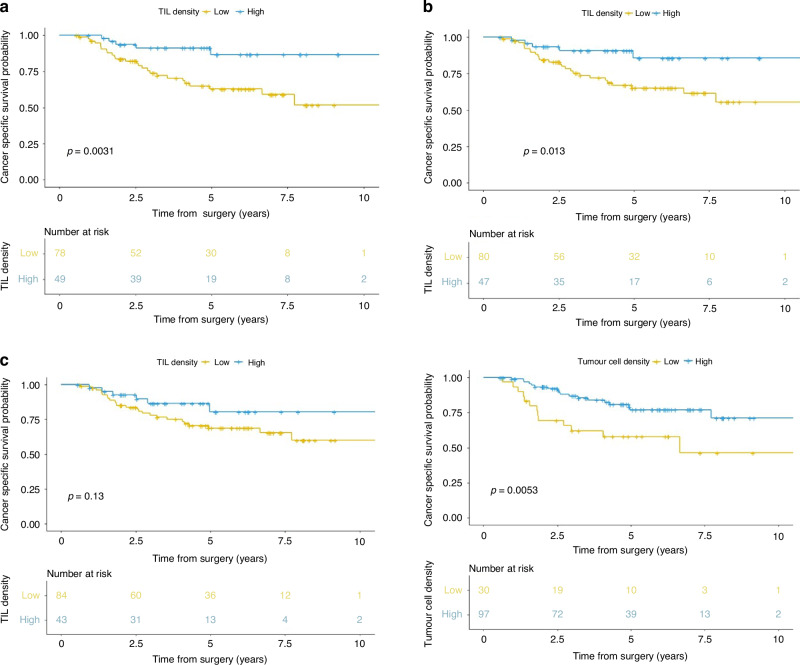
Cancer specific survival according to TIL density by annotation region and TCD. *P* value derived by the log rank test. **a** TIL at the invasive margin, **b** TIL across the whole tumour area, **c** TIL at the luminal surface, **d** TCD in a 3 × 3 mm box at the luminal surface. TIL tumour infiltrating lymphocytes, TCD tumour cell density.

TIL density was dichotomised in the whole tumour area (low ≤ 940 TILs/mm^2^, *n* = 80, high *n* = 47) the invasive margin (low ≤ 1155 TILs/mm^2^, *n* = 78, high *n* = 49), and the luminal surface (low ≤ 978 TILs/mm^2^, *n* = 84, high *n* = 43). Patients with low TIL density at the invasive margin or across the whole tumour area had poorer cancer-specific survival than those with high TIL density (HR 3.83, 95% CI 1.47-9.98, *P* = 0.006; HR 3.16, 95% CI 1.21–8.22, *P* = 0.019, respectively (Table 2, and Fig. 1a, b)). TIL density at the luminal surface was not significantly associated with survival (HR 1.96, 95% CI 0.81–4.80, *P* = 0.138; Fig. 1c). When only pMMR cases were analysed (*n* = 106), low TIL density at the invasive margin and across the whole tumour remained significantly associated with poorer cancer-specific survival than those with high TIL density (HR 4.58 95% CI 1.37–15.33, *P* = 0.013; HR 3.11, 95% CI 1.07–9.07, *P* = 0.019, respectively).

**Table 2:** Univariate and multivariate survival analysis for all clinicopathological variables

	All cases		Univariate cox regression		Multivariate cox regression^a^	
			Hazard ratio (95% CI)	*p*-value	Hazard ratio (95% CI)	*p*-value
	*n*	%				
**Sex**						
Male	50	39.4	1	–		
Female	77	60.6	0.66 (0.33–1.35)	0.26		
**Age (years)**						
<65	42	33.1	1	–		
≥65	85	66.9	1.80	0.17		
**Tumour location**						
Colon	89	70.1	1	–		
Rectum	38	29.9	1.09 (0.51–2.32)	0.81		
**Adjuvant chemotherapy**						
No	107	84.3	1	–		
Yes	20	15.7	1.43 (0.58–3.49)	0.44		
**pT category**^**b**^						
pT1/pT2	31	24.4	1	–	1	–
pT3	87	68.5	1.38 (0.52–4.68)	0.52	0.74 (0.26–2.09)	0.567
pT4	9	7.1	7.46 (2.26–24.61)	**<0.001**	3.15 (0.86–12.30)	0.081
**pN category**^**b**^						
pN0	83	65.4	1	–	1	–
pN1	28	22.0	1.86 (0.82–4.21)	0.138	1.42 (0.62–3.27)	0.411
pN2	16	12.6	2.94 (1.15–7.55)	**0.025**	3.33 (1.24–8.98)	**0.017**
**TNM stage**^**b**^						
I	26	20.5	1	–		
II	56	44.1	0.85 (0.30–2.46)	0.77		
III	44	34.6	1.91 (0.69–5.26)	0.21		
IV	1	0.8	∞	0.98		
**Distant metastasis**						
No	126	99.2	1	–		
Yes	1	0.8	∞	0.99		
**Lymphovascular invasion**						
No	122	96.1	1	–	1	–
Yes	5	3.9	4.13 (1.24–13.76)	**0.021**	5.95 (1.61–22.09)	**0.008**
**MMR status**						
pMMR	106	83.5	1	–		
dMMR	17	13.4	1.36 (0.56–3.30)	0.50		
NK	4	3.1				
**TILs invasive margin (per mm**^**2**^**)**						
Low ≤ 1155	78	61.4	3.83 (1.47–9.98)	**0.006**	4.28 (1.87–11.71)	**0.004**
High > 1155	49	38.6	1	–	1	–
**TILs whole tumour (per mm**^**2**^**)**						
Low ≤ 940	80	63.0	3.16 (1.21–8.22)	**0.019**		
High > 940	47	37.0	1	–		
**TILs luminal surface (per mm**^**2**^**)**						
Low ≤ 978	84	66.1	1.96 (0.81–4.80)	0.138		
High > 978	43	33.9	1	–		
**TCD luminal surface**						
Low (≤47%)	30	23.6	2.66 (1.30–5.42)	**0.007**	2.72 (1.19–6.17)	**0.017**
High (>47%)	97	76.4	1	–	1	–
**Combined TILs score at invasive margin and TCD**						
Low TCD and low TILs	15	11.8	1.0	–		
Low TCD and high TILs	15	11.8	0.11 (0.25–0.51)	0.005		
High TCD and low TILs	63	49.6	0.23 (0.10–0.49)	<0.001		
High TCD and high TILs	34	26.8	0.076 (0.02–0.27)	<0.001		
**Combined TCD and TILs score luminal surface**						
Low TCD and low TILs	17	13.4	1.0	–		
Low TCD and high TILs	13	10.2	∞	–		
High TCD and low TILs	67	52.8	0.14 (0.06–0.31)	<0.001		
High TCD and high TILs	30	23.6	0.21 (0.08–0.56)	0.002		

*P*-values that are statistically significant are shown in bold

*pT* depth of invasion, *pN* lymph node status, *pMMR* proficient mismatch repair, *dMMR* deficient mismatch repair, *NK* not known, *TILs* tumour infiltrating lymphocytes, *TCD* tumour cell density, *IM* invasive margin.

^a^Multivariate Cox model was adjusted for pT, pN, lymphovascular invasion, TILs at the invasive margin and TCD luminal surface.

^b^Tumour-Node-Metastasis stage grouping, pT category, pN category was obtained using TNM, 5^th^ edition [38].

TCD at the luminal surface was dichotomised at 47% (low *n* = 30, high *n* = 97). There was no relationship between TCD and any clinicopathological variables, including MMR status (Supplementary Table S2). Patients with low TCD had significantly poorer cancer-specific survival than those with high TCD (HR 2.66, 95% CI 1.30–5.42, *P* = 0.007, Fig. 1d). When only pMMR cases were analysed (*n* = 106), low TCD remained significantly associated with poorer cancer-specific survival than those with high TCD (HR 2.23, 95% CI 1.00–4.97, *P* = 0.049).

When TIL density at the invasive margin and TCD at the luminal surface were included in a multivariate model along with pT, pN and lymphovascular invasion, both low TIL density and low TCD were significantly associated with poorer cancer-specific survival (HR 4.28, 95% CI 1.87–11.71, *P* = 0.004; HR 2.72, 95% CI 1.19–6.17, *P* = 0.017, respectively) when compared to patients with high TIL density and high TCD (Table 2).

When TILs in the whole tumour area was included in the model instead of TILs at the invasive margin, both low TIL density and low TCD remained significantly associated with cancer-specific survival (HR 3.74, 95% CI 1.34–10.48, *P* = 0.012; HR 2.80, 95% CI 1.22–6.39; *P* = 0.015, respectively) when compared to those with high TIL density and high TCD (Supplementary Table S4). Survival analysis for data censored at 5 years is provided in Supplement.

### Combined TCD and TILs score and survival

**Fig. 2:**
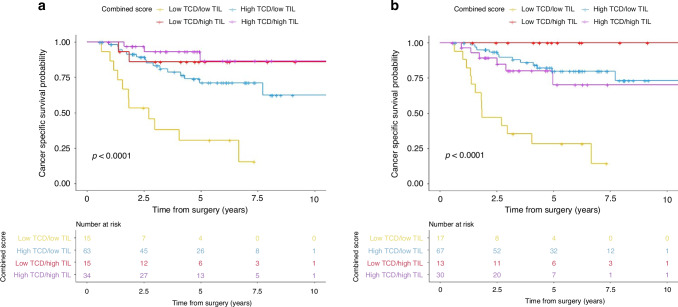
Cancer specific survival according to combined TIL density and TCD score by annotated region. *P* value derived by the log rank test. **a** TIL at the invasive margin and TCD at the luminal surface, **b** Both TIL and TCD at the luminal surface. TIL tumour infiltrating lymphocytes, TCD tumour cell density, IM invasive margin, LS luminal surface.

Correlation between TILs at the invasive margin and TCD was explored and no correlation was found (Pearson correlation coefficient: -0.149, *P* = 0.094). When the dichotomised scores for TILs at the invasive margin and TCD were combined, patients in the high TCD and TILs had a significantly greater cancer-specific survival than those with low TCD and TILs group (HR 0.08, 95% CI 0.02–0.27, *P* = < 0.001; Table 2, Fig. 2a); this remained significant on multivariate analysis (HR 0.10, 95% CI 0.03–0.40, *P* = 0.001; Supplementary Table S5). Those in the other two groups also had a significantly greater cancer-specific survival compared to those in the low TCD and TILs group (Supplementary Table S5).

When a further combined score was determined based on the dichotomised scores for both TCD and TILs at the luminal surface, patients in the high TCD and TILs group had a significantly greater cancer-specific survival compared to those in the low TCD and TILs group (HR 0.21 95% CI 0.08–0.56, *P* = 0.002; Fig. 2b). This remained significant on multivariate analysis (HR 0.29, 95% CI 0.10–0.81, *P* = 0.019; Supplementary Table S6).

## Discussion

A significant proportion of patients diagnosed with CRC will ultimately develop incurable distant metastatic disease [8], however, the identification of patients with high-risk disease who are most likely to benefit from adjuvant/neoadjuvant treatment remains challenging. There is therefore a pressing clinical need to develop novel prognostic biomarkers to aid decision making and to better identify this subset of patients. We aimed to explore two recognised but currently underutilised prognostic markers, TILs and TCD, both derived by DL on HE slides. To date, most studies on TILs have focused on the immune infiltrate at the deep invasive margin, which is notably defined differently across various studies, with some focussing on the infiltrate in the central tumour region instead [11, 13, 16]. There has been little focus on the immune infiltrate at the luminal surface, which is surprising given that this area is sampled in diagnostic biopsies and therefore opens the possibility for being used in decision making for neoadjuvant treatment.

Our study compared TIL density in different tumour regions of interest and aimed to explore the combined measurement of TILs and TCD as a prognostic marker, particularly at the luminal surface, to understand whether measuring these markers in biopsies could be a valid approach. The prospective assessment of TCD in different regions of the tumour is not the focus of this study, but will be explored in the future.

This study has shown that TIL density at the invasive margin is significantly greater compared to that across the whole tumour area and at the luminal surface. This is not unexpected as the invasive margin is considered to be the interface between tumour and normal tissue and therefore the primary site of interaction between malignant and immune cells. Many existing classification systems assess TILs at the invasive margin; similarly the presence of a “Crohn’s-like reaction” is noted at the deep aspect of the tumour [10–12, 18].

As expected from the current literature, high TIL density at both the invasive margin and across the whole tumour area was independently associated with greater cancer-specific survival on multivariate analysis [9, 16]. Similarly a high TCD was associated with greater cancer-specific survival on multivariate analysis. When dichotomised scores for TCD at the luminal surface and TIL density at the invasive margin were combined, patients with a low score in both had the worst cancer-specific survival when compared to those with a high scores in both. This demonstrates that the combined TCD and TILs score was a stronger prognostic marker than that of TILs or TCD in isolation. When looking at the results for the other two groups, it appeared that TIL density at the invasive margin had a greater impact on survival than TCD at the luminal surface with the low TCD and high TIL group showing a similar hazard ratio to that of the high TCD and high TIL group.

As a single marker, TIL density at the luminal surface was not significantly associated with prognosis, but there was a trend between high TILs and improved cancer-specific survival as seen in other areas of the tumour. Given the size of the hazard ratio, this likely reflects a type 2 error and this analysis may well be significant in a larger series of patients. As expected from current literature, this study found that TCD at the luminal surface was an independent prognostic marker in CRC. In contrast to other studies that have largely focused on the invasive margin [29, 31–33], this measure was assessed at the luminal surface of the tumour in keeping with the previous work we have performed using manual assessment [28]. When TCD and TIL density scores from the luminal surface were combined, the combined score was a stronger prognostic marker than that of TCD in isolation; patients with low scores in both had the worst cancer-specific survival when compared to those with high scores in both.

The luminal surface of a resection specimen is essentially the same region sampled in a diagnostic endoscopic biopsy, with the depth of the luminal strip in our study chosen to replicate the depth of a typical biopsy sample. These prognostic scores are easy to obtain and could be combined with existing clinicopathological factors to augment clinical decision making; the luminal surface scoring in particular has the potential to be applied to biopsy specimens to identify the subset of patients with the worst outcomes and even influence neoadjuvant decision making. Biopsy samples offer unique challenges in comparison to resection samples in that they can be heterogeneous and are frequently small and fragmented. Despite this, they are increasingly being used for molecular biomarker testing and therefore decision making for neoadjuvant treatment. The composition of immune cells and TCD in biopsy samples has been found to be important by others [19, 43]. Studies utilising IHC to identify specific T-cell subsets in biopsies from patients with locally advanced rectal cancer prior to neoadjuvant chemo-radiotherapy have found high Immunoscore in the pre-treatment biopsy was associated with improved 5 year recurrence rates compared to low Immunoscore; however, these results are only from a subset of cases showing a complete response to neoadjuvant therapy [20, 21]. These studies rely on IHC and little work has been done on TIL detection on HE in CRC biopsies to date. The value of manually measuring TCD on biopsy samples has been demonstrated in oesophageal adenocarcinoma with low TCD associated with worse overall survival; however, there was significant inter-observer variation in TCD scoring between pathologists [43]. Low TCD in CRC biopsies has also been found to be significantly associated with the rate of lymph node metastasis in potentially curable CRC patients [44].

TCD and TILs have previously been combined to create a composite score called the Glasgow Microenvironment Score; this score has been found to be a prognostic biomarker in stage I-III CRC, however this system requires manual assessment of both TCD and TILs [37]. Another study in CRC has combined DL tumour stroma estimations and DL immune scores and found a beneficial effect of a combined score [35]; this study however looked at TCD and immune scores within the invasive margin component only; our study also considers the use of a combined TCD and TIL density score at the luminal surface.

To date, both TILs and TCD are not used in routine clinical practice largely due to the time-consuming nature of manual scoring by pathologists and also the subjective nature of such assessments. Measuring TILs and TCD by DL is objective, cheap and unlike Immunoscore can be carried out on HE without requiring additional tissue sections to be cut and stained from the block [16]. Digital scanning is increasingly used in routine clinical practice internationally and a move to DL assessment could save significant resources in a financially stretched health service. DL has been used to separately quantify TILs in the stromal and epithelial compartments on HE in triple negative breast cancer with high TILs in both regions associated with improved prognosis [22]. Quantifying TILs into epithelial and stromal compartments is therefore recommended in breast cancer management pathways [45], however there are no similar recommendations in CRC at the present time. Another study demonstrated that DL TIL quantification was both prognostic and predictive of response to adjuvant chemotherapy in stage II-III gastric cancer; similarly to our study, this work did not separate TILs into epithelial or stromal compartments [24]. DL has been used in CRC to detect “Crohn’s-like reaction” density at the invasive margin, where greater levels were also associated with improved prognosis, however, in contrast to our study, this methodology used an area based convolutional neural network instead of UNET based cell detection [46]. UNET based cell detection identifies and segments individual cells rather than being based on pixels, and may therefore be superior at calculating TIL density [47].

The current study has some limitations. This study used the 5^th^ edition of the TNM classification [38] compared to the 8^th^ edition used in current practice [48]. Serial changes to TNM staging could impact the multivariate analyses used in our study. We appreciate that the small size of this cohort and lack of a validation cohort is a significant limitation of this study in respect to validation of our cut-off values. However, this combined biomarker approach will be investigated in the recently published international FOxTROT trial of advanced but operable colon cancer, including a straight to surgery group (*n* = 354) and a neoadjuvant chemotherapy group (*n* = 699) [49], where diagnostic biopsy samples are also available alongside surgical resections.

In conclusion, we have explored the prognostic value of TIL density in different regions of the tumour along with TCD at the luminal surface. We have shown that luminal surface TCD and invasive margin TIL density measured on HE using DL based methods are both independently prognostic in CRC. Patients with combined low TCD and TIL score are at highest risk of death. The DL methodology for both biomarkers is cheap, objective, should be more reproducible and quicker to perform and could be used as an adjunct alongside routine biomarkers to stratify and identify those patients who may benefit from adjuvant therapy. TCD and TIL density when both measured at the luminal surface have the potential to be used on biopsy samples to aid prognostication in the neoadjuvant setting. Further studies are in progress to validate this work in an independent cohort and to investigate the prognostic value of TCD and TIL density in biopsy samples from patients with CRC. As TCD and TIL density have been found to be prognostic in other solid tumour types, this DL based method and combined biomarker approach should also be explored in other solid tumour types.

## Supplementary information

Supplementary Information

## Data Availability

The data that support the findings of this study are not openly available but are available from the corresponding author upon reasonable request. Data are located in controlled access data storage at the University of Leeds.
