# Supplementary material for: Deep-learning enabled combined measurement of tumour cell density and tumour infiltrating lymphocyte density as a prognostic biomarker in colorectal cancer
Source: BJC Rep. 2025 Mar 3;3:12. doi: 10.1038/s44276-025-00123-8 (PMC11876445; doi:10.1038/s44276-025-00123-8)
Supplement: Supplementary file 1 — Supplementary Information [file 44276_2025_123_MOESM1_ESM.docx]

**Supplementary information**

**Supplementary data**

**Figure S1:** Example of annotations on haematoxylin-eosin (HE) stained colorectal cancer resection whole slide image

**Figure S2:** Examples of HE images with corresponding DL segmentation results for TIL density and TCD

**Figure S3:** Study flow chart

**Figure S4:** 5 year cancer specific survival according to TIL density by annotation region and TCD

**Figure S5:** 5 year cancer specific survival according to combined TIL density and TCD score by annotated region

**Table S1:** REporting recommendations for tumour MARKer prognostic studies (REMARK)

**Table S2:** Relationship between TCD and clinicopathological variables

**Table S3** Univariate and multivariate survival analysis for all clinicopathological variables censored at 5 years

**Table S4:** Multivariate analysis including TILs in the whole tumour

**Table S5:** Multivariate analysis with combined TCD and TILs at the invasive margin score

**Table S6:** Multivariate analysis with combined TCD and TILs at the luminal surface score

**Table S7:** Multivariate analysis censored at 5 years including TILs in the whole tumour

**Table S8:** Multivariate analysis censored at 5 years with combined TCD and TILs at the invasive margin score

**Table S9:** Multivariate analysis censored at 5 years with combined TCD and TILs at the luminal surface score

**Figure S1.** Example of annotations on haematoxylin-eosin (HE) stained colorectal cancer resection whole slide image. **A)** Annotation of the whole tumour area. **B)** Annotation of the luminal surface strip (2mm in depth from the luminal surface). **C)** Annotation of the invasive margin strip (1mm in depth into the tumour from the tumour/normal interface).


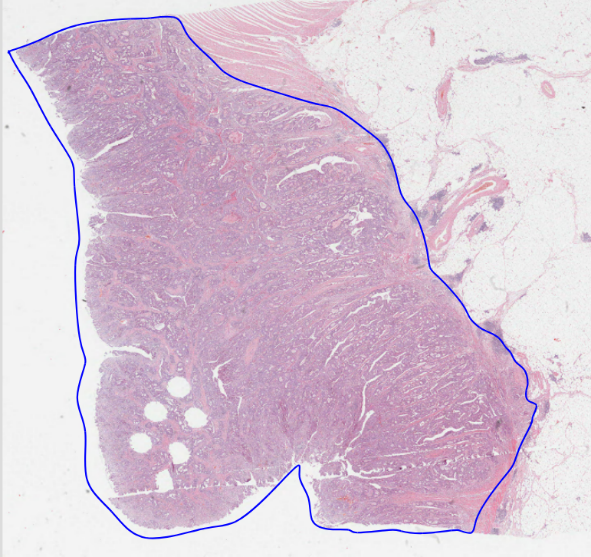

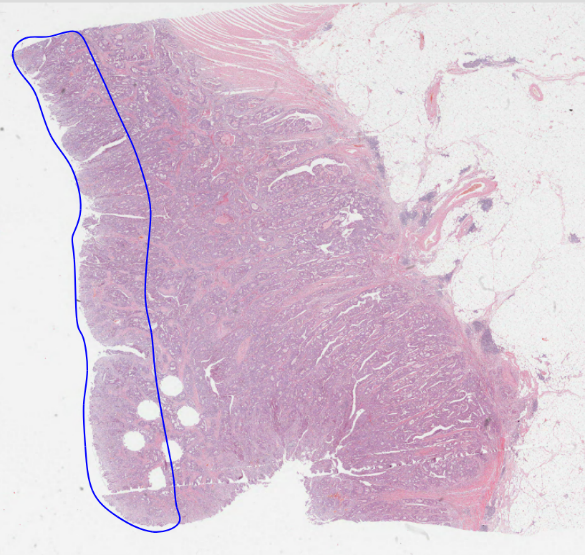

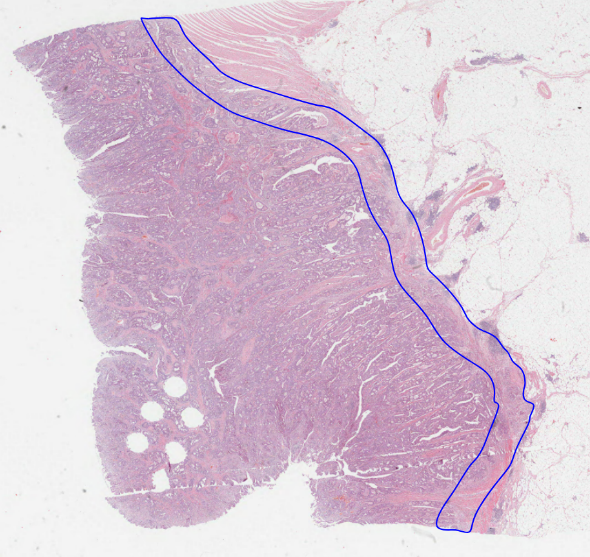


**A**

**B**

**C**

**Figure S2.** Examples of HE images with corresponding DL segmentation results for TIL density and TCD. **A & B**. Examples of low and high TIL density respectively. Taken at 20 x magnification. Red segmentation = tumour infiltrating lymphocytes. **C & D**. Examples of low and high TCD respectively taken at 5 x magnification. Green segmentation = tumour, blue segmentation = stroma, orange segmentation = non-informative point, yellow segmentation = necrosis, light-blue segmentation = tumour lumen, pink segmentation = inflammation.

HE = haematoxylin and eosin; TCD = tumour cell density; TIL = tumour infiltrating lymphocyte; DL = deep-learning


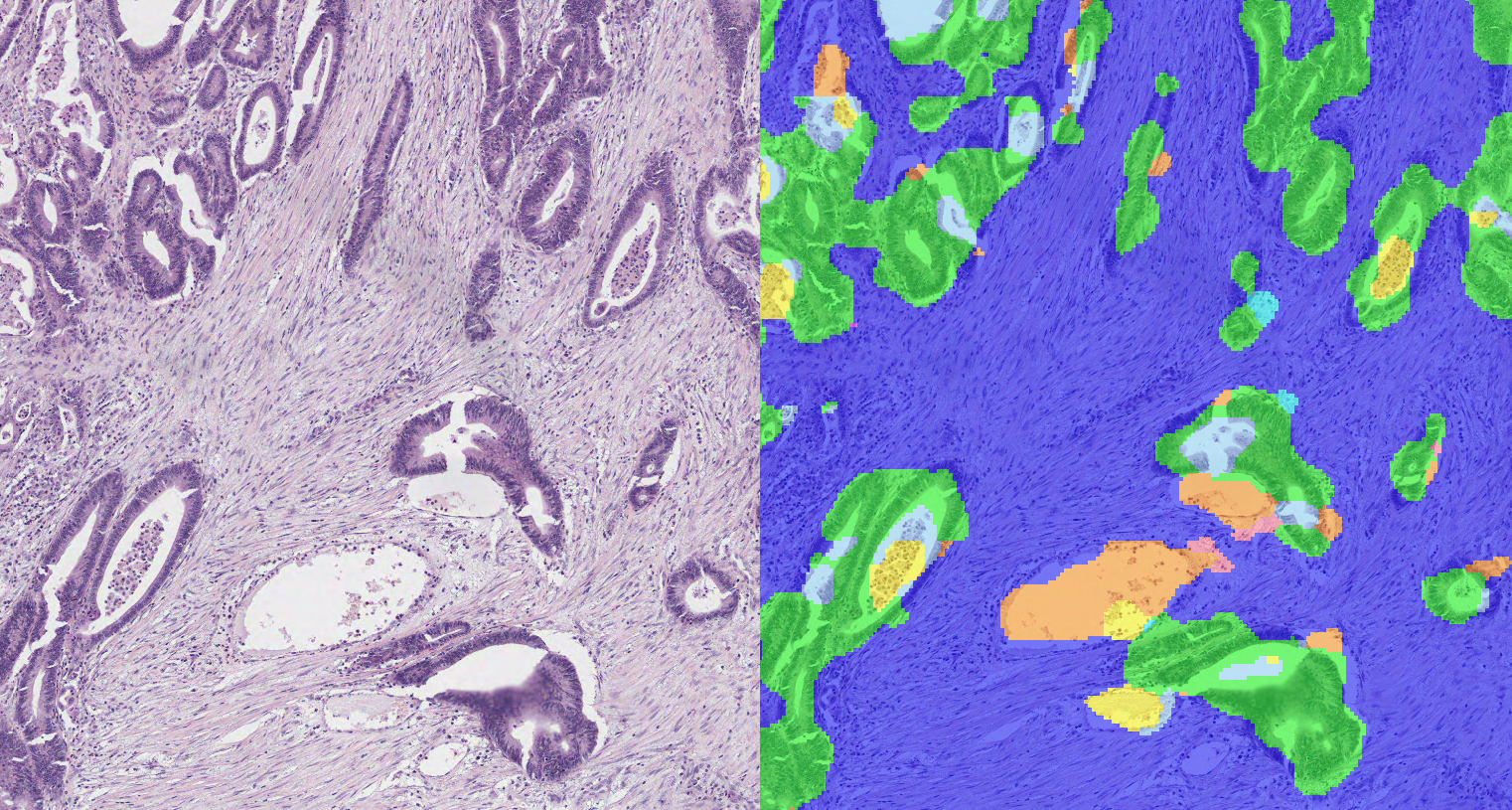

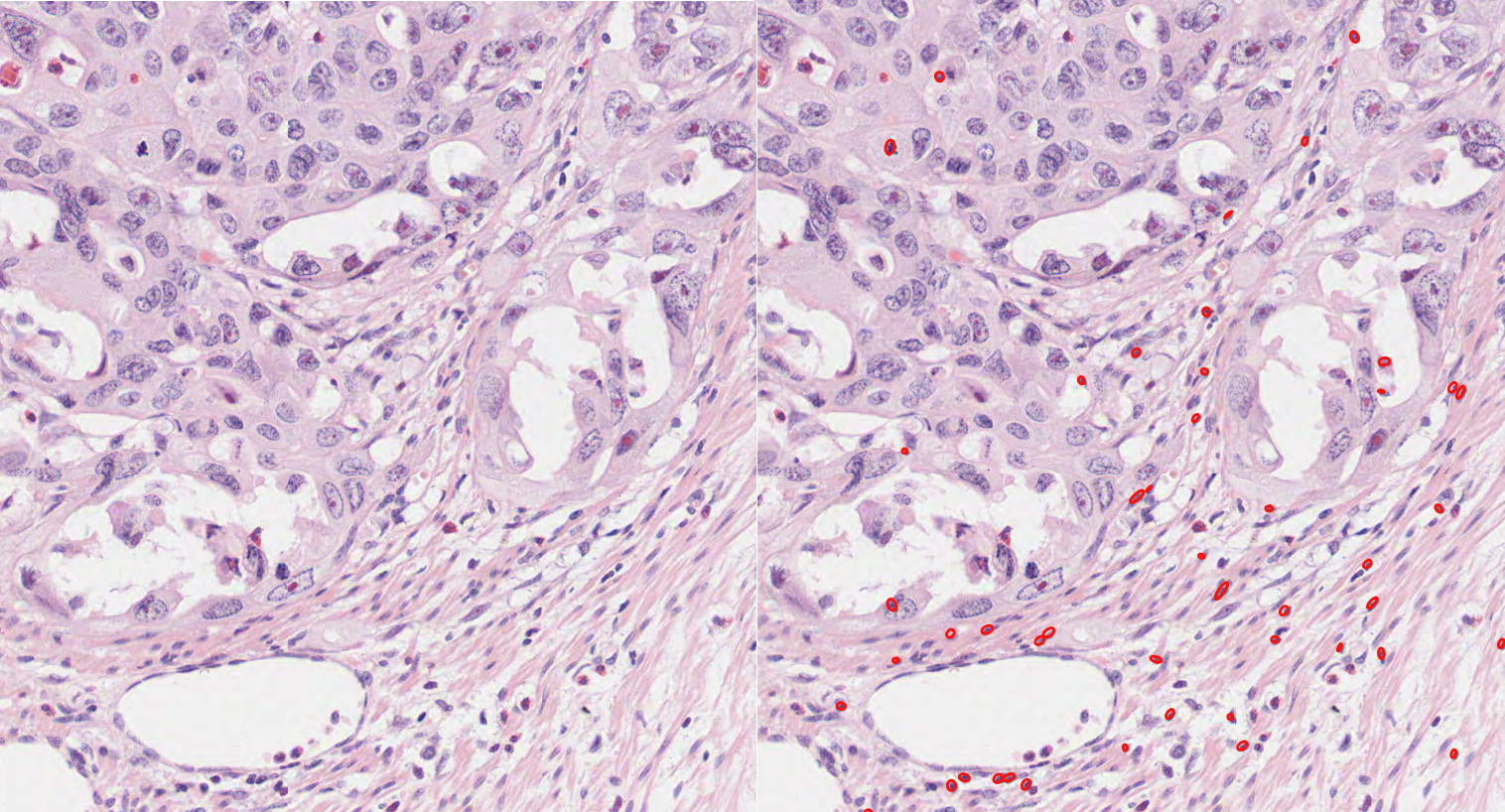


C


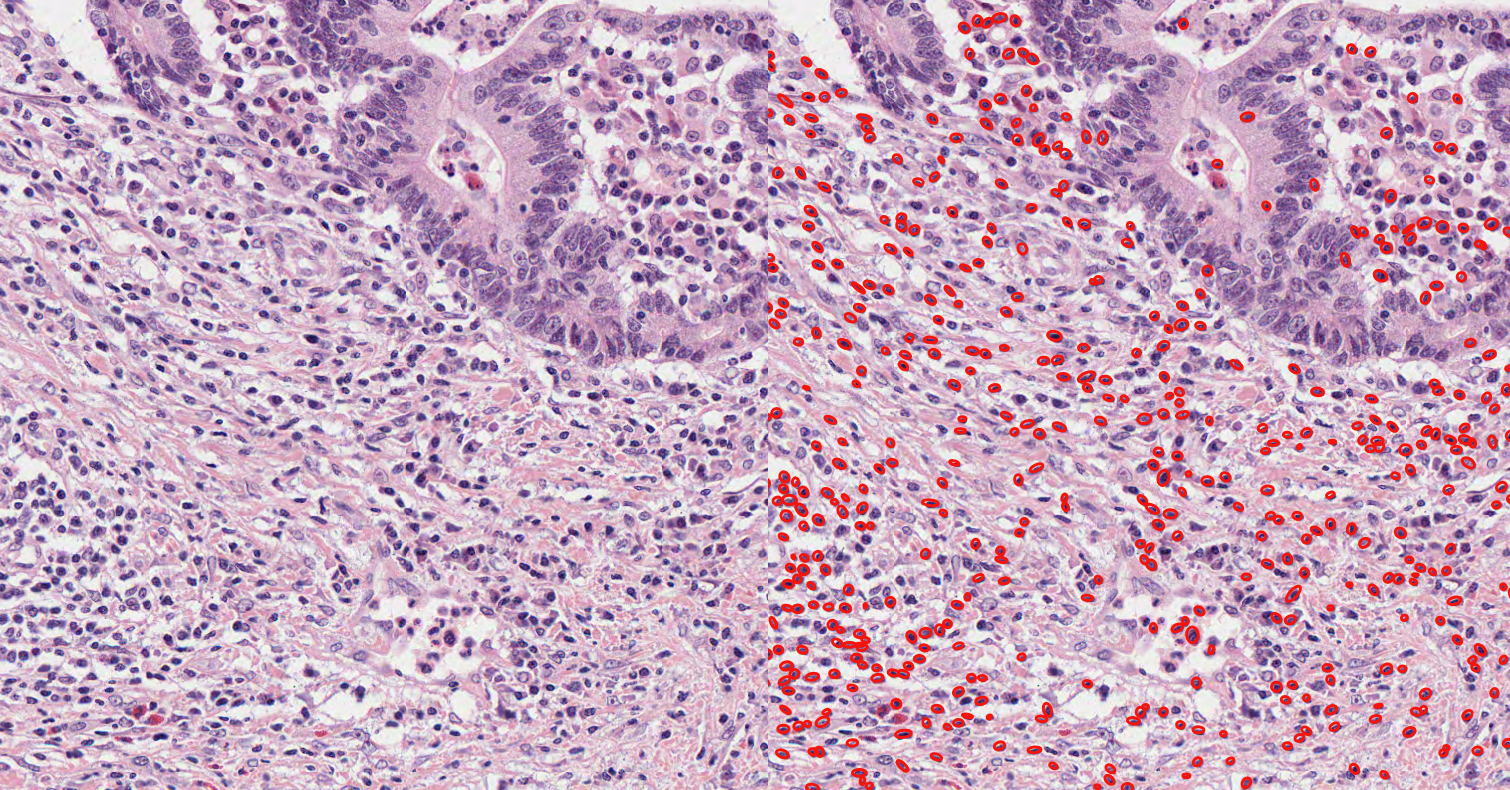


A


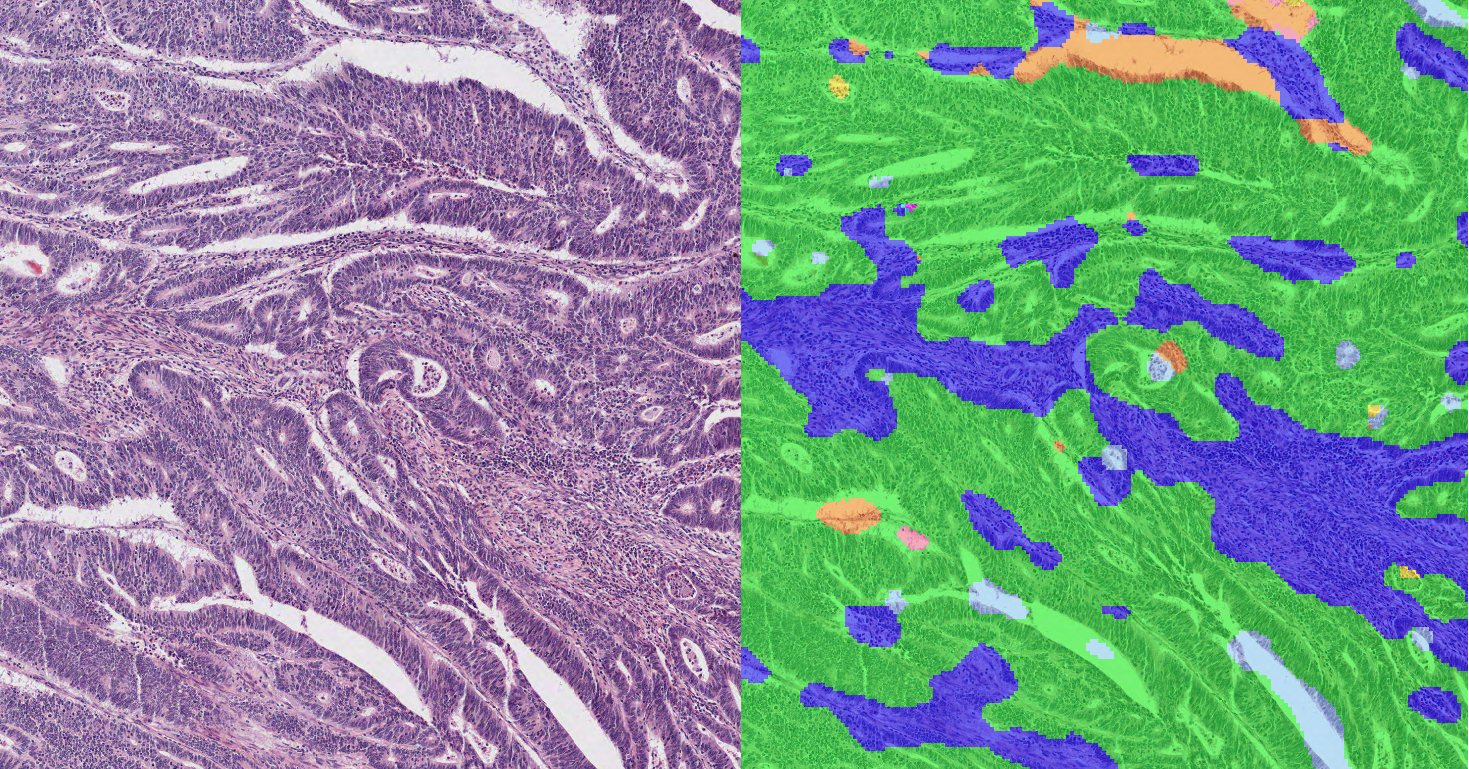


B

D

**Figure S3.** Study flow chart

144 digital WSI of CRCs (one per patient) with manual TCD result

140 cases annotated with whole tumour region

Excluded n = 4:

- n= 3 poor adherence of tissue to slide
- n=1 polyp with no definite area of invasion

Invasive strip annotated in 134 cases

127 cases with all three regions annotated

Excluded n = 7:

- n= 3 no visible luminal surface
- n= 4 poor adherence of tissue at the luminal surface

Excluded n = 6:

- n= 3 polyps with no invasive margin visible due to orientation
- n=3 poor adherence of tissue at the invasive margin

WSI = whole slide image, CRC = colorectal cancer, TCD = tumour cell density

**Figure S4**. 5 year cancer specific survival according to TIL density by annotation region and TCD. P value derived by the log rank test. **A**. TIL at the invasive margin, **B.** TIL across the whole tumour area, **C.** TIL at the luminal surface, **D.** TCD in a 3x3 mm box at the luminal surface


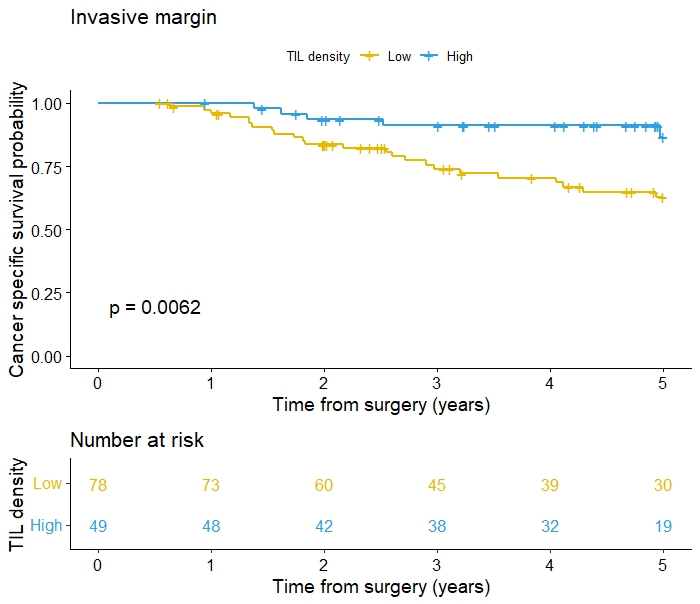


**A**


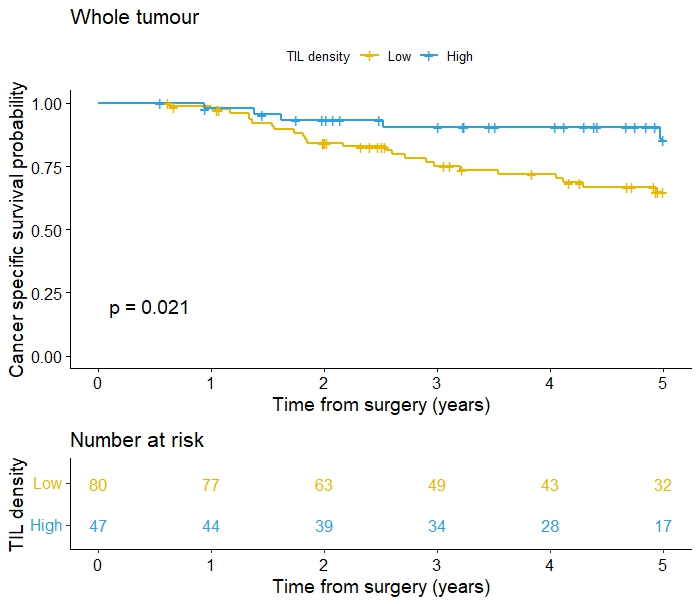


**B**


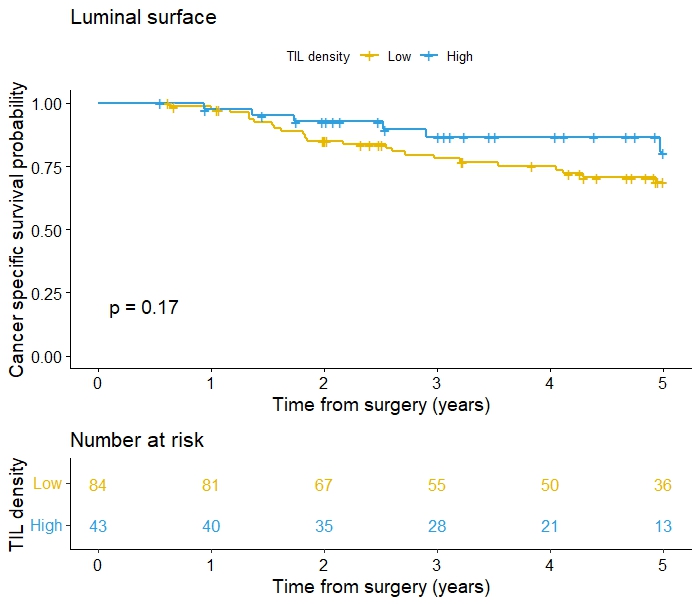


**C**


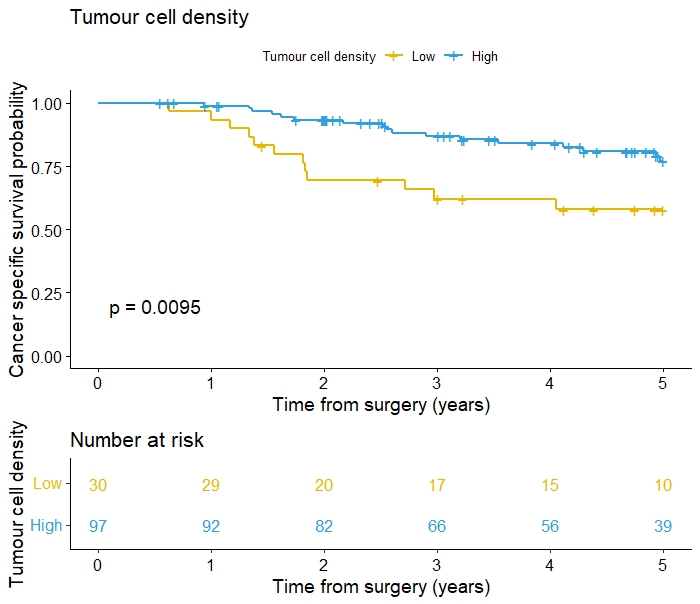


**D**


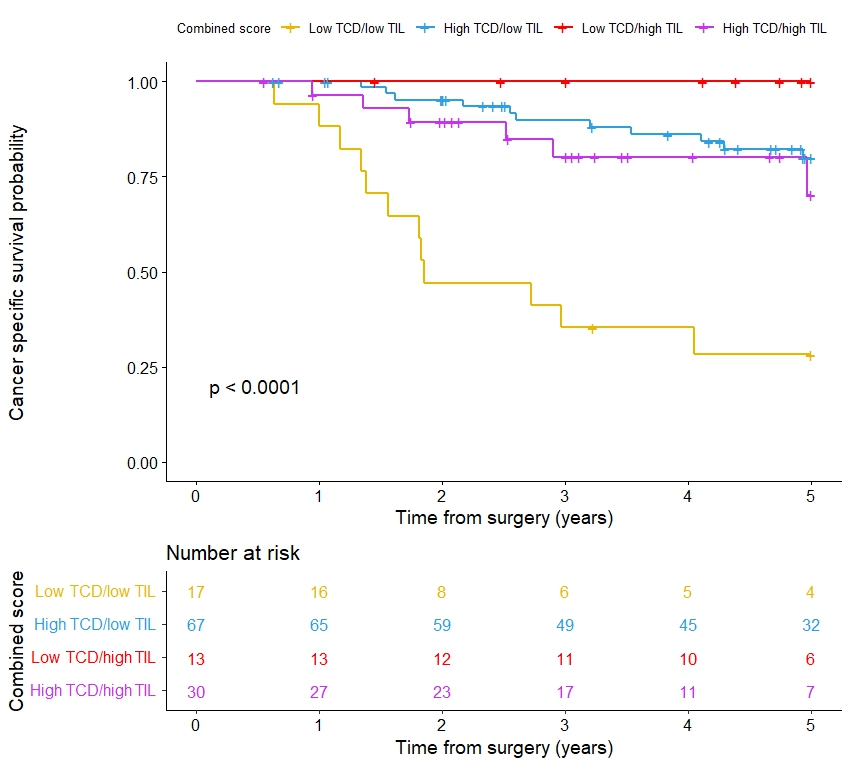
**
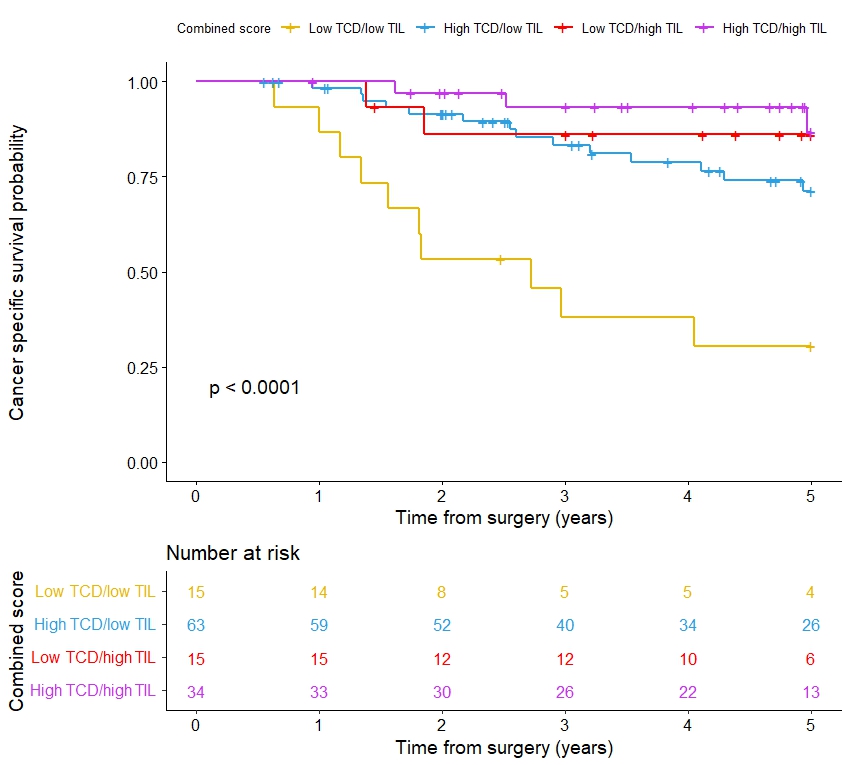
Figure S5.** 5 year cancer specific survival according to combined TIL density and TCD score by annotated region. P value derived by the log rank test. **A**. TIL at the invasive margin and TCD at the luminal surface, **B.** Both TIL and TCD at the luminal surface

**B**

**A**

| **Number (REMARK)** | **Reference to Main Manuscript & Comment** |
| --- | --- |
| 1.: study objectives, hypotheses | Last sentence of background: 1. high TIL density is associated with improved survival on univariate and multivariate analysis, 2. Combining TIL and TCD values improved prognostication in CRC, 3. combining TILs and TCD values derived from the luminal surface improves prognostication |
| 2./3.: patients: characteristics and treatments | Methods ('Patients and clinicopathological data'). 20 patients received adjuvant therapy (no precise treatment regimens available). Patients treated with neoadjuvant therapy were excluded. |
| 4.: specimen characteristics | Methods ('Patients and clinicopathological data'). CRC resection specimens, stained with H&E, scanned at 40x. |
| 5.: assay methods | Methods (''Image analysis pipeline to establish TIL density” and “Image analysis pipeline to establish TCD”). Description of the model used to detect TILs can be found in (23) and in supplementary methodology. |
| 6.-9.: study design | Methods ('Patients and clinicopathological data'). Retrospective study of consecutive cases without matching or randomization (between January 1990 and December 1995); endpoints: cancer-specific survival. |
| 10./11.: statistical analysis methods | Methods ('Statistical Analyses'). |
| 12./13.: data | Study flow chart is depicted in Figure S2. All Kaplan-Meier curves are provided with risk tables. Clinicopathological characteristics are provided in Table 1. |
| 14./15.: analysis and presentation | Relation of TIL density to standard prognostic variables is shown in Table 1. Univariate and multivariate Cox Regression Analysis is provided in Table 2, Tables S1-S7. Kaplan-Meier plots are given in Figure 1, Figure 2, S3, and S4. Confidence intervals as well as p-values are reported. |
| 19./20.: discussion | Limitations of the current study are discussed in the final paragraphs of the discussion. Future implications of our work are also provided. Further studies are in progress to validate this work in an independent cohort and to investigate the prognostic value of TCD and TIL density in biopsy samples from patients with CRC. Our findings with regards to the use of TCD and TIL density when both measured at the luminal surface have the potential to be used on biopsy samples to aid prognostication in the neoadjuvant setting. |
| CRC: colorectal cancer, H&E: haematoxylin and eosin, TIL: tumour infiltrating lymphocyte, TCD: tumour cell density | |

**Table S1**. REporting recommendations for tumour MARKer prognostic studies (REMARK)

**Table S2.** Relationship between TCD and clinicopathological variables

|  | | **All cases** | | **Tumour cell density** |
| --- | --- | --- | --- | --- |
|  | | **n** | **%** | **p-value** |
| **Sex** |  |  |  |  |
|  | Male | 50 | 39.4 | 0.25 |
|  | Female | 77 | 60.6 |  |
| **Age (years)** |  |  |  |  |
|  | <65 | 42 | 33.1 | 0.93 |
|  | ≥65 | 85 | 66.9 |  |
| **Tumour location** | |  |  |  |
|  | Colon | 89 | 70.1 | 0.14 |
|  | Rectum | 38 | 29.9 |  |
| **Adjuvant chemotherapy** | |  |  |  |
|  | No | 107 | 84.3 | 0.98 |
|  | Yes | 20 | 15.7 |  |
| **pT category^a^** | |  |  |  |
|  | pT1/pT2 | 31 | 24.4 | 0.22 |
|  | pT3 | 87 | 68.5 |  |
|  | pT4 | 9 | 7.1 |  |
| **pN category^a^** | |  |  |  |
|  | pN0 | 83 | 65.4 | 0.42 |
|  | pN1 | 28 | 22.0 |  |
|  | pN2 | 16 | 12.6 |  |
| **TNM stage^a^** | |  |  |  |
|  | I | 26 | 20.5 | 0.48 |
|  | II | 56 | 44.1 |  |
|  | III | 44 | 34.6 |  |
|  | IV | 1 | 0.8 |  |
| **Distant metastasis** | |  |  |  |
|  | No | 126 | 99.2 | 0.58 |
|  | Yes | 1 | 0.8 |  |
| **Lymphovascular invasion** | |  |  |  |
|  | No | 122 | 96.1 | 0.99 |
|  | Yes | 5 | 3.9 |  |
| **Mismatch repair status** | |  |  |  |
|  | pMMR | 106 | 83.5 | 0.16 |
|  | dMMR | 17 | 13.4 |  |
|  | NK | 4 | 3.1 |  |

**Table S3**. Univariate and multivariate survival analysis for all clinicopathological variables censored at 5 years

|  | | **All cases** | | **Univariate cox regression** | | **Multivariate cox regression^a^** | |
| --- | --- | --- | --- | --- | --- | --- | --- |
|  |  |  |  | **Hazard ratio (95%CI)** | **p-value** | **Hazard ratio (95%CI)** | **p-value** |
|  |  | **n** | **%** |  |  |  |  |
| **Sex** | |  |  |  |  |  |  |
|  | Male | 50 | 39.4 | 1 | - |  |  |
|  | Female | 77 | 60.6 | 0.68 (0.33-1.42) | 0.31 |  |  |
| **Age (years)** | |  |  |  |  |  |  |
|  | <65 | 42 | 33.1 | 1 | - |  |  |
|  | ≥65 | 85 | 66.9 | 1.62 (0.69-3.79) | 0.27 |  |  |
| **Tumour location** | |  |  |  |  |  |  |
|  | Colon | 89 | 70.1 | 1 | - |  |  |
|  | Rectum | 38 | 29.9 | 0.89 (0.40-2.02) | 0.79 |  |  |
| **Adjuvant chemotherapy** | |  |  |  |  |  |  |
|  | No | 107 | 84.3 | 1 | - |  |  |
|  | Yes | 20 | 15.7 | 1.49 (0.61-3.66) | 0.39 |  |  |
| **pT category^b^** | |  |  |  |  |  |  |
|  | pT1/pT2 | 31 | 24.4 | 1 | - | 1 | - |
|  | pT3 | 87 | 68.5 | 1.65 (0.56-4.86) | 0.36 | 0.89 (0.29-2.79) | 0.84 |
|  | pT4 | 9 | 7.1 | 9.58 (2.68-34.22) | **<0.001** | 4.24 (1.03-17.43) | **0.045** |
| **pN category^b^** | |  |  |  |  |  |  |
|  | pN0 | 83 | 65.4 | 1 | - | 1 | - |
|  | pN1 | 28 | 22.0 | 2.13 (0.92-4.92) | 0.08 | 1.63 (0.69-3.84) | 0.26 |
|  | pN2 | 16 | 12.6 | 3.45 (1.32-9.02) | **0.011** | 3.75 (1.36-10.31) | **0.010** |
| **TNM stage^b^** | |  |  |  |  |  |  |
|  | I | 26 | 20.5 | 1 | - |  |  |
|  | II | 56 | 44.1 | 0.98 (0.31-3.13) | 0.97 |  |  |
|  | III | 44 | 34.6 | 2.44 (0.81-7.35) | 0.11 |  |  |
|  | IV | 1 | 0.8 | ∞ | 0.98 |  |  |
| **Distant metastasis** | |  |  |  |  |  |  |
|  | No | 126 | 99.2 | 1 | - |  |  |
|  | Yes | 1 | 0.8 | ∞ | 0.70 |  |  |
| **Lymphovascular invasion** | |  |  |  |  |  |  |
|  | No | 122 | 96.1 | 1 | - | 1 | - |
|  | Yes | 5 | 3.9 | 4.13 (1.24-13.77) | **0.021** | 5.96 (1.61-22.10) | **0.008** |
| **MMR status** | |  |  |  |  |  |  |
|  | pMMR | 106 | 83.5 | 1 | - |  |  |
|  | dMMR | 17 | 13.4 | 1.17 (0.16-8.68) | 0.88 |  |  |
|  | NK | 4 | 3.1 |  |  |  |  |
| **TILs invasive margin (per mm^2^)** | |  |  |  |  |  |  |
|  | Low ≤1,155 | 78 | 61.4 | 3.53 (1.35-9.25) | **0.010** | 3.55 (1.30-9.71) | **0.014** |
|  | High >1,155 | 49 | 38.6 | 1 | - | 1 | - |
| **TILs whole tumour (per mm^2^)** | |  |  |  |  |  |  |
|  | Low ≤ 940 | 80 | 63.0 | 2.96 (1.13-7.75) | **0.027** |  |  |
|  | High > 940 | 47 | 37.0 | 1 | - |  |  |
| **TILs luminal surface (per mm^2^)** | |  |  |  |  |  |  |
|  | Low ≤ 978 | 84 | 66.1 | 1.86 (0.77-4.58) | 0.18 |  |  |
|  | High > 978 | 43 | 33.9 | 1 | - |  |  |
| **TCD luminal surface** | |  |  |  |  |  |  |
|  | Low (≤47%) | 30 | 23.6 | 2.57 (1.23-5.38) | **0.012** | 2.33 (0.99-5.46) | 0.051 |
|  | High (>47%) | 97 | 76.4 | 1 | - | 1 | - |
| **Combined TILs score at invasive margin and TCD** | |  |  |  |  |  |  |
|  | Low TCD and low TILs | 15 | 11.8 | 1.0 | - |  |  |
|  | Low TCD and high TILs | 15 | 11.8 | 0.13 (0.03-0.61) | **0.009** |  |  |
|  | High TCD and low TILs | 63 | 49.6 | 0.25 (0.11-0.55) | **<0.001** |  |  |
|  | High TCD and high TILs | 34 | 26.8 | 0.09 (0.02-0.32) | **<0.001** |  |  |
| **Combined TCD and TILs score luminal surface** | |  |  |  |  |  |  |
|  | Low TCD and low TILs | 17 | 13.4 | 1.0 | - |  |  |
|  | Low TCD and high TILs | 13 | 10.2 | ∞ | 0.97 |  |  |
|  | High TCD and low TILs | 67 | 52.8 | 0.15 (0.07-0.34) | **<0.001** |  |  |
|  | High TCD and high TILs | 30 | 23.6 | 0.23 (0.09-0.62) | **0.003** |  |  |

*P*-values that are statistically significant are shown in bold

^a^ Multivariate Cox model was adjusted for pT, pN, lymphovascular invasion, TILs at the invasive margin and TCD luminal surface.

^b^ Tumour-Node-Metastasis stage grouping, pT category, pN category was obtained using TNM, 5^th^ edition (38).

pT = depth of invasion, pN = lymph node status, pMMR = proficient mismatch repair, dMMR = deficient mismatch repair, NK = not known, TILs = tumour infiltrating lymphocytes, TCD = tumour cell density, IM = invasive margin

**Table S4.** Multivariate analysis including TILs in the whole tumour

|  | | **Multivariate cox regression^a^** | |
| --- | --- | --- | --- |
|  |  | **Hazard ratio (95%CI)** | **p-value** |
| **pT stage** |  |  |  |
|  | pT1/pT2 | 1 | - |
|  | pT3 | 0.72 (0.25-2.05) | 0.53 |
|  | pT4 | 3.20 (0.84-12.18) | 0.089 |
| **pN stage** |  |  |  |
|  | pN0 | 1 | - |
|  | pN1 | 1.50 (0.65-3.43) | 0.34 |
|  | pN2 | 3.68 (1.36-9.97) | **0.010** |
| **Lymphovascular invasion** | |  |  |
|  | No | 1 | - |
|  | Yes | 4.95 (1.36-17.99) | **0.015** |
| **TCD luminal surface** | |  |  |
|  | Low (≤47%) | 2.80 (1.22-6.39) | **0.015** |
|  | High (>47%) | 1 | - |
| **TILs whole tumour (per mm^2^)** | |  |  |
|  | Low ≤ 940 | 3.74 (1.33-10.48) | **0.012** |
|  | High > 940 | 1 | - |

*P*-values that are statistically significant are shown in bold.

^a^ Multivariate Cox model was adjusted for pT, pN, lymphovascular invasion, TCD luminal surface and TILs whole tumour

pT = depth of invasion, pN = lymph node status, TILs = tumour infiltrating lymphocytes, TCD = tumour cell density

**Table S5.** Multivariate analysis with combined TILs at the invasive margin and TCD score

|  | | **Multivariate cox regression^a^** | |
| --- | --- | --- | --- |
|  |  | **Hazard ratio (95%CI)** | **p-value** |
| **pT stage** |  |  |  |
|  | pT1/pT2 | 1 | - |
|  | pT3 | 0.76 (0.26-2.16) | 0.60 |
|  | pT4 | 3.04 (0.80-11.60) | 0.10 |
| **pN stage** |  |  |  |
|  | pN0 | 1 | - |
|  | pN1 | 1.43 (0.92-3.30) | 0.41 |
|  | pN2 | 5.57 (1.48-20.98) | **0.015** |
| **Lymphovascular invasion** | |  |  |
|  | No | 1 | - |
|  | Yes | 5.57 (1.48-20.98) | **0.011** |
| **Combined TCD and TILs score invasive margin** | |  |  |
|  | Low TCD and low TILs | 1.0 | - |
|  | Low TCD and high TILs | 0.16 (0.03-0.78) | **0.024** |
|  | High TCD and low TILs | 0.32 (0.13-0.80) | **0.015** |
|  | High TCD and high TILs | 0.10 (0.03-0.40) | **0.001** |

*P*-values that are statistically significant are shown in bold.

^a^ Multivariate Cox model was adjusted for pT, pN, lymphovascular invasion and combined TCD and TILs score.

pT = depth of invasion, pN = lymph node status, TILs = tumour infiltrating lymphocytes, TCD = tumour cell density

**Table S6.** Multivariate analysis with combined TCD and TILs at the luminal surface score

|  | | **Multivariate cox regression** | |
| --- | --- | --- | --- |
|  |  | **Hazard ratio (95%CI)** | **p-value** |
| **pT stage** |  |  |  |
|  | pT1/pT2 | 1 | - |
|  | pT3 | 0.89 (0.32-2.49) | 0.83 |
|  | pT4 | 2.21 (0.58-8.37) | 0.24 |
| **pN stage** |  |  |  |
|  | N0 | 1 | - |
|  | N1 | 1.40 (0.60-3.27) | 0.44 |
|  | N2 | 3.54 (1.31-9.58) | **0.013** |
| **Lymphovascular invasion** | |  |  |
|  | No | 1 | - |
|  | Yes | 4.92 (1.34-18.05) | **0.016** |
| **Combined TCD and TILs score luminal surface** | |  |  |
|  | Low TCD and low TILs | 1.0 | - |
|  | Low TCD and high TILs | ∞ | 0.98 |
|  | High TCD and low TILs | 0.16 (0.07-0.41) | **<0.001** |
|  | High TCD and high TILs | 0.29 (0.10-0.81) | **0.019** |

*P*-values that are statistically significant are shown in bold.

^a^ Multivariate Cox model was adjusted for pT, pN, lymphovascular invasion and combined TCD and TILs score at the luminal surface.

pT = depth of invasion, pN = lymph node status, TILs = tumour infiltrating lymphocytes, TCD = tumour cell density

|  | | **Multivariate cox regression^a^** | |
| --- | --- | --- | --- |
|  |  | **Hazard ratio (95%CI)** | **p-value** |
| **pT stage** |  |  |  |
|  | pT1/pT2 | 1 | - |
|  | pT3 | 0.88 (0.28-2.76) | 0.82 |
|  | pT4 | 4.20 (1.01-17.43) | **0.048** |
| **pN stage** |  |  |  |
|  | pN0 | 1 | - |
|  | pN1 | 1.73 (0.74-4.05) | 0.21 |
|  | pN2 | 4.12 (1.49-11.38) | **0.006** |
| **Lymphovascular invasion** | |  |  |
|  | No | 1 | - |
|  | Yes | 5.04 (1.38-18.41) | **0.014** |
| **TCD luminal surface** | |  |  |
|  | Low (≤47%) | 2.38 (1.01-5.59) | **0.048** |
|  | High (>47%) | 1 | - |
| **TILs whole tumour (per mm^2^)** | |  |  |
|  | Low ≤ 940 | 3.10 (1.10-8.69) | **0.033** |
|  | High > 940 | 1 | - |

**Table S7**. Multivariate analysis censored at 5 years including TILs in the whole tumour

*P*-values that are statistically significant are shown in bold.

^a^ Multivariate Cox model was adjusted for pT, pN, lymphovascular invasion, TCD luminal surface and TILs whole tumour

pT = depth of invasion, pN = lymph node status, TILs = tumour infiltrating lymphocytes, TCD = tumour cell density

**Table S8**. Multivariate analysis censored at 5 years with combined TILs at the invasive margin and TCD score

|  | | **Multivariate cox regression^a^** | |
| --- | --- | --- | --- |
|  |  | **Hazard ratio (95%CI)** | **p-value** |
| **pT stage** | |  |  |
|  | pT1/pT2 | 1 | - |
|  | pT3 | 0.92 (0.29-2.88) | 0.88 |
|  | pT4 | 3.98 (0.96-16.55) | 0.06 |
| **pN stage** | |  |  |
|  | pN0 | 1 | - |
|  | pN1 | 1.65 (0.70-3.88) | 0.26 |
|  | pN2 | 3.93 (1.42-10.91) | **0.009** |
| **Lymphovascular invasion** | |  |  |
|  | No | 1 | - |
|  | Yes | 5.58 (1.48-21.05) | **0.011** |
| **Combined TILs score at the invasive margin and TCD** | |  |  |
|  | Low TCD and low TILs | 1.0 | - |
|  | Low TCD and high TILs | 0.20 (0.04-0.98) | **0.047** |
|  | High TCD and low TILs | 0.37 (0.14-0.98) | **0.044** |
|  | High TCD and high TILs | 0.14 (0.03-0.56) | **0.006** |

*P*-values that are statistically significant are shown in bold.

^a^ Multivariate Cox model was adjusted for pT, pN, lymphovascular invasion and combined TCD and TILs score.

pT = depth of invasion, pN = lymph node status, TILs = tumour infiltrating lymphocytes, TCD = tumour cell density

**Table S9.** Multivariate analysis censored at 5 years with combined TCD and TILs at the luminal surface score

|  | | **Multivariate cox regression** | |
| --- | --- | --- | --- |
|  |  | **Hazard ratio (95%CI)** | **p-value** |
| **pT stage** |  |  |  |
|  | pT1/pT2 | 1 | - |
|  | pT3 | 1.06 (0.35-3.26) | 0.92 |
|  | pT4 | 2.98 (0.72-12.45) | 0.13 |
| **pN stage** |  |  |  |
|  | N0 | 1 | - |
|  | N1 | 1.63 (0.69-3.88) | 0.27 |
|  | N2 | 4.16 (1.50-11.52) | **0.006** |
| **Lymphovascular invasion** | |  |  |
|  | No | 1 | - |
|  | Yes | 5.03 (1.36-18.53) | **0.015** |
| **Combined TCD and TILs score luminal surface** | |  |  |
|  | Low TCD and low TILs | 1 | - |
|  | Low TCD and high TILs | ∞ | 0.98 |
|  | High TCD and low TILs | 0.20 (0.08-0.52) | **<0.001** |
|  | High TCD and high TILs | 0.36 (0.12-1.04) | 0.06 |

*P*-values that are statistically significant are shown in bold.

^a^ Multivariate Cox model was adjusted for pT, pN, lymphovascular invasion and combined TCD and TILs score at the luminal surface.

pT = depth of invasion, pN = lymph node status, TILs = tumour infiltrating lymphocytes, TCD = tumour cell density
